# Pregnancy prediction via ultrasound-detected endometrial blood for hormone replacement therapy-frozen embryo transfer: a prospective observational study

**DOI:** 10.1186/s12958-023-01164-9

**Published:** 2023-11-24

**Authors:** Xue Ke, Xue-fei Liang, Yong-hong Lin, Fang Wang

**Affiliations:** Reproductive Center Chengdu Women’s and Children’s Central Hospital, School of Medicine, University of Electronic Science and Technology of China, 1617 Riyue Avenue, Chengdu, 611731 Sichuan China

**Keywords:** Power doppler ultrasound, Endometrial receptivity, Endometrial blood flow, Frozen embryo transfer

## Abstract

**Background:**

This study aimed to assess the predictive value of endometrial blood flow branches on pregnancy outcomes after hormone replacement therapy-frozen embryo transfer (HRT-FET).

**Methods:**

This prospective observational study involved 292 reproductive-aged women who underwent endometrial receptivity assessment in a tertiary care academic medical center in southwest China using power Doppler ultrasonography during HRT-FET. Three-dimensional power Doppler ultrasound was performed on the day of endometrial transformation and the day before embryo transfer. The endometrial blood flow branches of the endometrial and subendometrial regions were compared in the non-pregnant and pregnant groups at the two time points mentioned above.

**Results:**

The endometrial blood flow branches were higher in pregnant patients than in non-pregnant patients on the day of endometrial transformation (P = 0.009) and the day before embryo transfer (P = 0.001). Changes in endometrial blood flow pattern and endometrial blood flow branches at the two time points did not differ among the pregnancy outcome samples. After adjusting for age, antral follicles, and embryos transferred, the endometrial blood flow branches on the day before embryo transfer was the independent factor influencing the chance of clinical pregnancy, with an odds ratio of 3.001 (95% confidence interval: 1.448 − 6.219, P = 0.003).

**Conclusions:**

Endometrial blood flow perfusion during the peri-transplantation period of the HRT-FET cycle is a good indicator of pregnancy outcomes, suggesting that valuation of endometrial branches via power Doppler ultrasound is a simple and effective approach for achieving indicator measurements.

## Background

Accurate assessment of endometrial development and receptivity remains a challenge in reproductive medicine. None of the endometrial receptivity markers that can be evaluated by endometrial biopsy, endometrial fluid aspiration, or hysteroscopy have sufficient discriminatory value to act as a diagnostic measure for endometrial receptivity [1]. However, ultrasound remains the most non-invasive and reproducible method for evaluating endometrial receptivity compared to invasive procedures, such as histology and molecular research. Previous studies have focused on the effects of independent ultrasound parameters on embryo implantation, including endometrial patterns and thickness, which have been repeatedly tested and compared with pregnancy rates during in vitro fertilization (IVF) cycles with conflicting results [2, 3]. In recent years, some studies have focused on the comprehensive evaluation of ultrasound parameters for predicting endometrial receptivity, including conservative endometrial measurements, endometrial myometrial junction studies, and comprehensive scores of endometrial and subendometrial Doppler indices [4, 5], which are considered to represent better the actual ability of the endometrium to accept embryos.

Among the various ultrasound parameters, endometrial blood flow perfusion is typically considered necessary for embryo implantation. This factor can be non-invasively evaluated and roughly estimated using color Doppler ultrasound; however, power Doppler imaging is more sensitive for detecting low-speed flow, with stronger visibility of small blood vessels, increased accuracy in counting, and high operability [6, 7]. Therefore, power Doppler has the potential to become a daily detection tool for checking the blood supply to the entire endometrium and subendometrial area.

To the best of our knowledge, research on endometrial blood flow correlating power Doppler ultrasound (PD-US) data with hormone replacement therapy-frozen embryo transfer (HRT-FET) pregnancy outcomes remains rare, and the optimal measurement time for pregnancy outcome prediction remains unclear. Moreover, in most independent or comprehensive parameters used to evaluate endometrial receptivity, the evaluation is often limited to a specific time point, such as oocyte retrieval day and embryo transfer day. In this study, we evaluated the role of endometrial receptivity ultrasound parameters in predicting pregnancy on the day of endometrial transformation and the day before embryo transfer in HRT-FET cycles. At the same time, endometrial and subendometrial blood flow changes at two time points were added to evaluate the prediction performance. These results may be used to assess the value of incorporating appropriate endometrial receptivity indicators into routine monitoring during HRT-FET cycles.

## Methods

### Study subjects

This prospective observational study was conducted at a tertiary care academic medical center in southwestern China. Women who received their first HRT-FET between November 2021 and July 2023 were invited to participate in this study. Based on previous ultrasound, hysterosalpingography, magnetic resonance imaging, or surgical records, patients with stage III and IV endometriosis, adenomyosis, hydrosalpinx, uterine fibroids, history of severe uterine adhesions, endometrial polyps, scarred uterus, or uterine malformations were excluded. Furthermore, patients with a history of recurrent miscarriage or repeated implantation failure (more than three occurrences) were excluded from the study. This study was approved by the Scientific Ethics Committee of Chengdu Women’s and Children’s Central Hospital (reference: B2021-7), and all enrolled patients provided informed consent.

### FET procedures

For hormonal replacement therapy cycles, all patients received estradiol (Femoston red tablets; Abbott Biologicals B.V., Olst, NL) treatment at 4 mg orally, with the dosage regulated on a 7-day basis according to endometrial thickness (EMT). If the EMT was < 8 mm, the estradiol dosage was increased. When the EMT reached ≥ 8 mm, and the oral estradiol dosage reached ≥ 14 days, or the clinicians and patients recognized EMT as having a relatively maximum thickness, progesterone was administered to initiate endometrial transformation. The secretory transformation was initiated using fematon-yellow tablets (containing estradiol 2 mg and dydrogesterone 10 mg; Abbott Biologicals B.V.) twice daily, and 40 mg oil-based intramuscular progesterone injection (Zhejiang Xianju Pharmaceutical) once daily was administered from the day of transformation to the day of the pregnancy test.

### Power Doppler operation and data collection

Identity 3D power Doppler (3D PD-US, H60, Samsung, Seoul, South Korea) settings with a transvaginal probe of 4 − 9 MHz were used in patients for endometrial receptivity testing on the endometrial transformation day as well as on the day preceding embryo transfer at approximately 1 pm. The settings for this study were as follows: low frequency; signal magnifying, 50; pulse repetition frequency, 0.8 kHz; power Doppler map, 5; and power, 90%. The sector of interest was adjusted to cover the endometrial cavity in the longitudinal plane of the uterus. The color gain was adjusted to 80%±2% to optimize blood flow detection in the small vessels of endometrial and subendometrial areas.

**Fig. 1:**
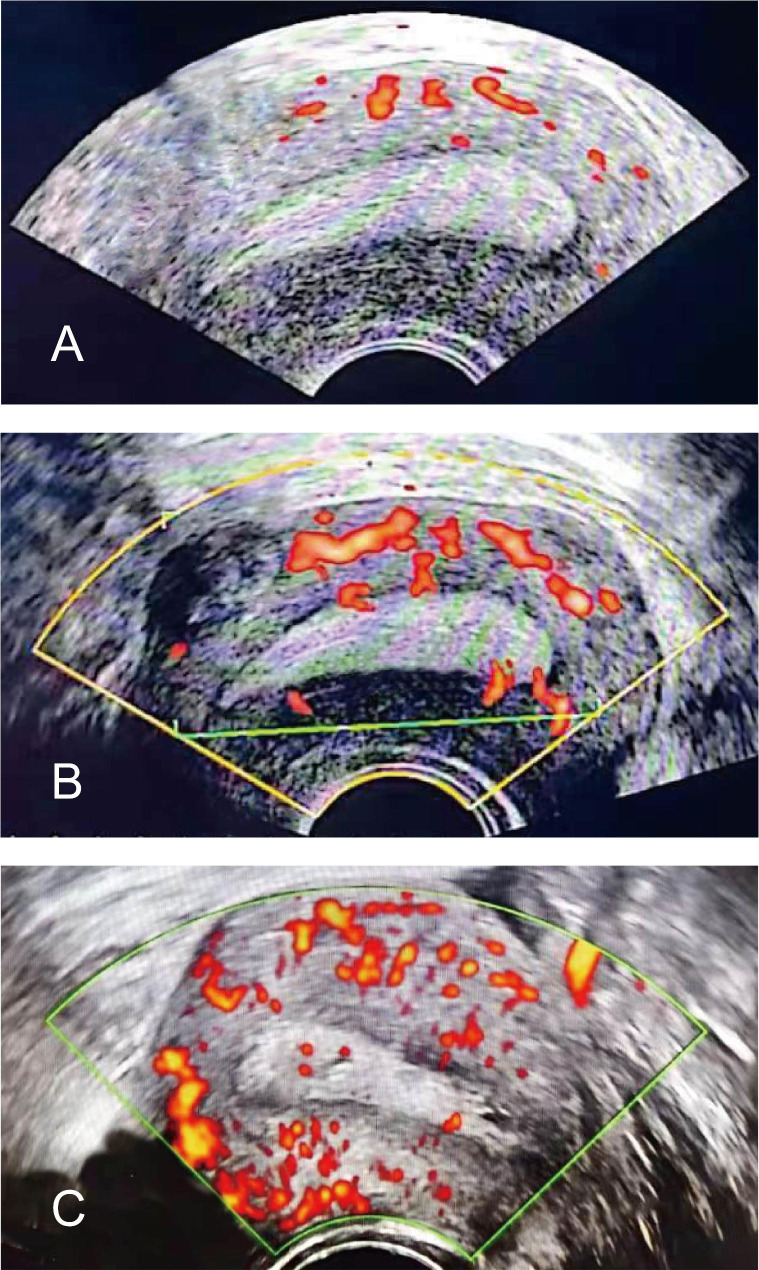
Power Doppler ultrasound imaging of endometrial and subendometrial blood flow: type-branch. **(A)** type I - branch<5: the vessels pass through the lateral hypo-echoic band of the endometrium but do not enter the hyper-echoic rim of the endometrium, branch 1. **(B)** type II - branch<5: the vessels pass through the hyper-echoic rim of the endometrium but do not enter the endometrium, branch 4. **(C)** type III – branch <5: the vessels enter the endometrium, branch 4

The data produced by the PD-US measurement included EMT, endometrial patterns (classified as type A, trilinear endometrium; type Not-A, no trilinear endometrium), endometrial contraction, endometrial volume, blood flow patterns (type I, the vessels pass through the lateral hypo-echoic band of the endometrium but do not enter the hyper-echoic rim of the endometrium; type II, the vessels pass through the hyper-echoic rim of the endometrium but do not enter the endometrium; type III, the vessels enter the endometrium [8]), and endometrial blood flow branches (countable endometrial and subendometrial blood flow branches in a single plane) (Fig. 1). In this study, the subendometrial region was considered to be within 1 mm of the originally defined myometrial–endometrial contour [9]. The endometrial volume was drawn manually along the endometrial outline. Stored volumes were analyzed using VOCAL software of Three-dimensional (3D) Power Doppler. The results of the ultrasound evaluation did not affect the subsequent clinical procedures. The primary outcome measure was intrauterine clinical pregnancy (defined as the presence of a gestational sac in the uterus determined using ultrasonography).

The Kappa test was applied to evaluate the consistency of the endometrial blood flow pattern results, with a Kappa value of 0.778 (P = 0.001). The Kendall W test was used to evaluate the consistency of the endometrial blood flow branches, with the Kendall Wa being 0.979 (P = 0.001). An ROC curve was constructed to explore the cutoff value of EMBLB for Pregnancy outcome.

### Statistical analyses

Statistical analyses were performed using the Statistical Program for Social Sciences (SPSS Version 26, IBM Corp, Armonk, NY, USA). Normal distribution was identified using the Shapiro-Wilk test. Data were statistically described in terms of mean ± standard deviation (± SD), median and range, or number of cases (frequencies) when appropriate normality of data was tested. A nonparametric Mann-Whitney U test was used to compare the non-normally distributed data. Other comparisons were performed using the chi-squared and Fisher’s Exact tests, where appropriate. Binary stepwise logistic regression was used to determine the independent predictive factor of clinical pregnancy, adjusting for patient age, antral follicles (AFC), number of embryos transferred, embryo transfer type, good quality embryos transfer, endometrial blood flow, and changes of blood flow at two time points. The results are presented as the odds ratio (OR) and 95% confidence interval (CI). The significance level for all analyses was set at P < 0.05.

## Results

A total of 292 patients who underwent HRT-FET met the inclusion criteria and were processed for this study. They completed PD-US tests on the day of endometrial transformation and the day before embryo transfer. Among them, 176 patients achieved clinical pregnancy, including two who had a tubal pregnancy (2/176) and twelve who had spontaneous abortions (12/176). The remaining 116 patients were classified into the non-pregnant group, with an intrauterine pregnancy rate of 59.59% per HRT-FET. The demographic and clinical characteristics of the patients are presented in Table 1.

**Table 1:** Demographic and clinical characteristics of pregnant and non-pregnant patients undergoing HRT-FET

Characteristic	Pregnant group	Non-pregnant group	P-value
No. of patients	174	116	---
Age (years)	31.57 ± 4.41	33.07 ± 4.24	0.004
BMI (kg/m^2^)	21.80 (19.88 − 23.43)	22.60((20.03 − 24.00)	0.109
AMH (ng/mL)	3.20(1.68 − 5.84)	2.67(1.63 − 4.90)	0.101
AFC	12.0 (8.0 − 17.0)	10.0 (6.0 − 14.0)	0.004
Primary infertility (n, %)	90 (51.72)	59 (50.86)	0.886
Cause of infertility (n, %)			0.426
Tubal	118 (67.82)	81 (69.83)	
Aovulatory	8(4.60)	6 (5.17)	
DOR	17 (9.77)	16(13.79)	
Endometriosis Male	5 (2.87)	1(0.86)	
Unexplained	3 (1.72)	0 (0.00)	
Male factor	23 (13.22)	12 (10.34)	
No. of embryos transferred	1.56 ± 0.50	1.48 ± 0.50	0.180
Blastocysts transfer cycles (n, %)	107 (61.49)	60 (51.72)	0.099
Good quality embryos transfer cycles (n, %)	138 (79.31)	86 (74.14)	0.303

Values are means ± SD or number (percentage) of patients. Abbreviations: BMI body mass index; AMH anti-Müllerian hormone; AFC antral follicle; DOR Decreased ovarian reserve.

## Discussion

The results of this study demonstrated that measuring the endometrial blood perfusion using PD-US was a simple and effective method for predicting pregnancy outcomes during the HRT-FET cycle. Embryos are estimated to account for one-third of implantation failures, whereas poor endometrial receptivity and altered embryo endometrial dialogue account for the remaining two-thirds [10]. Several studies have examined EMT, endometrial type, subendometrial contraction, and endometrial volume and have attempted to provide a clear overview of the implantation window. However, contradictory findings have been reported [11, 12]. The ultrasound endometrial indicators in our study were not the final factors affecting pregnancy outcomes.

This study evaluated the role of PD-US in predicting pregnancy during HRT-FET cycles by counting endometrial and subendometrial blood flow branches. The pregnant group was younger and had higher AFC values. However, after adjusting for these parameters, the branches of the spiral arteries on the day before transplantation were the only factors that affected pregnancy outcomes. While Wang et al. concluded that the coexistence of endometrial and subendometrial blood flow results in a higher embryo implantation rate [13], our results suggest that blood perfusion is more critical than the endometrial − subendometrial blood flow distribution pattern. Blood supply to the endometrium comes from the radial artery, which passes through the connection between the uterine muscle and endometrium, divides into basal arteries that supply the basal layer, and then forms a spiral artery to reach the surface of the endometrium. At the junction of the myometrium and endometrium, ultrasonography shows a low-echo area called the subendometrial area. Histological studies have confirmed that this area is the innermost layer of the myometrium, or the junction zone [14]. Compared to the outer muscular layer, our results showed that blood flow in the junction zone was more abundant in most cases. Thus, whether the branch enters the endometrium the day before embryo transfer may not be an absolute factor affecting pregnancy outcomes; instead, the richness of blood flow in the endometrium or junction zone is suggested as a predictive factor.

In this study, all eight patients with endometrial blood flow branches > 10 on the progesterone start day were pregnant, while 13 with blood flow branches > 10 on the day before transplantation had conceived. Therefore, we emphasize the possible positive relationship between the abundance of blood flow during the secretory phase and pregnancy; however, it should not be concluded that patients without endometrial or subendometrial blood flow branches have absolutely low implantation and pregnancy rates. In patients with blood flow branch counts of 0 − 1 on either the day of transformation or the day before transplantation, we did not find a linear correlation between no blood flow and pregnancy. These data indicate that there may be a certain threshold for endometrial vascularization that, when reached, has a significant positive impact on clinical pregnancy. Whether the research conclusions can become a reference option for selective embryo transfer or guide clinical medication requires further large-scale prospective cohort studies. For patients with poor blood flow on the day of embryo transfer, can we improve pregnancy outcomes through drugs that increase local endometrial blood flow? The related drug effects will be announced in the future.

Ng et al. [15] reported that the measurement of endometrial and subendometrial blood flow using 3D PD-US could not predict pregnancy well if measured at a time point during IVF treatment. Due to baseline differences between patients in the present study, we compared the changes in 3D PD-US parameters of the endometrium at the two time points. However, we failed to confirm that the changes on the day of endometrial transformation and the day before embryo transfer significantly differed between the pregnant and non-pregnant groups. It has not been confirmed whether this change has any role in predicting HRT-FET results, and the main variable determining pregnancy outcomes is endometrial blood flow during the peri-transplantation period.

This study had the advantage of being the first prospective cohort study to use PD-US to detect endometrial receptive ultrasound indicators at two time points after HRT-FET. Due to the inconsistent Doppler signal waveforms of these small spiral vessels, predicting pregnancy using the spiral artery Doppler flow index is highly controversial [16–18]; thus, our study did not measure the subendometrial vascularization flow index but instead chose a more intuitive indicator for assessing blood perfusion. Previous studies have reported that blood flow to the endometrium affects pregnancy outcomes after freeze-thawed embryo transfer; however, the detection methods needed to be more consistent. Jinno et al. [19] measured endometrial tissue blood flow (ETBF) using laser blood flowmetry and found that women with an endometrial microvascular blood flow of at least 29 mL/min per 100 g of tissue in the early luteal phase had a significantly higher pregnancy rate, with 85% of gestational sacs in normal uteri implanted in the uterine regions with the highest measured ETBF. Other studies have demonstrated that vascular endothelial growth factor levels and its receptors significantly increase during the peri-implantation period [20–22]. Additionally, 3D Doppler angiography has been shown to quantitatively evaluate the vascular density and blood flow in the endometrium and subendometrium [23]. However, none of these methods have the operability of real-time monitoring during endometrial preparation for FET, whereas 3D PD-US can achieve the convenience of bedside monitoring.

There was a limitation to the current study. Most patients refused to undergo transvaginal ultrasound on the day of embryo transfer. Therefore, after endometrial transformation, we performed a second endometrial receptivity test in the afternoon before transplantation, and there may have been some deviations from the actual values on the day of transplantation.

## Conclusion

Measuring the endometrial and subendometrial blood flow branches on the day of the peri-transplantation period during the HRT-FET cycle using PD-US was shown to be a simple and effective method for predicting pregnancy outcomes. In the future, the development of ultrasound may improve the consistency of endometrial blood flow assessment and generate more accurate non-invasive blood flow assessment methods for daily clinical practice, such as microvascular flow ultrasound.

### Comparison of characteristics between pregnant and non-pregnant patients

There was a significant difference in age distribution between the pregnancy and non-pregnancy groups (**31.57 ± 4.41** years vs. **33.07 ± 4.24** years; P = **0.004**) as well as a significant difference in bilateral AFC between the two groups (**12.0 (8.0 − 17.0)** vs. **10.0 (6.0 − 14.0)**; **P = 0.004**). However, both groups did not show significant differences in body mass index, anti-Müllerian hormone levels, the proportion of primary infertility, and the cause of infertility. The fallopian tube was the most common infertility factor in both groups. The proportion of blastocyst transfer cycles (**61.49% vs. 51.72%**) and good-quality embryo transfer cycles (**79.31% vs. 74.14%**) was higher in the pregnancy group than in the non-pregnancy group, but without significant difference (Table 1).

### Endometrial receptivity indices on the day of endometrial transformation and the day before embryo transfer

Table 2 summarizes the endometrial PD-US indices on the days of endometrial transformation and before transplantation. An ROC curve was constructed to explore the predictive value of EMBLB for Pregnancy outcome, and to find the cut-off value [area under the curve (AUC)0.662, 95% CI 0.559–0.725, cutoff = 5].On the day of endometrial transformation, there were significant differences in blood flow branches among the different clinical pregnancy outcome samples (P = 0.009). On the day before transplantation, endometrial blood flow branches were higher in pregnant patients than in non-pregnant patients (P = 0.001). However, both groups were similar regarding EMT, frequency or direction of endometrial contraction, endometrial blood flow pattern, and endometrial volume at the two time points. All eight patients with endometrial blood flow branches ≥ 10 were pregnant on the day of secretory transformation. Meanwhile, there were 14 patients with endometrial blood flow branches > 10 on the day before transplantation, 13 were pregnant, and the remaining one who was not pregnant was transplanted with a D6 poor-quality blastocyst.

**Table 2:** Comparison of endometrial receptivity of 3D power Doppler indices on the day of endometrial transformation and the day before embryo transfer between pregnant and non-pregnant patients

Parameter	endometrial transformation day		P-value		the day before embryo transfer		P-value
	Pregnant	Non-pregnant group			Pregnant	Non-pregnant	
No. of patients	174	116	——		174	116	——
EMT (mm)	9.0 (8.0 − 11.0)	9.0 (8.0 − 10.5)	0.878		9.0 (8.0 − 11.0)	9.0 (8.0 − 10.5)	0.458
EMP (n,%)			0.518				0.713
A	113 (64.9)	71(61.2)			20 (11.5)	15 (12.9)	
N-A	61 (35.1)	45 (38.8)			154 (88.5)	101 (87.1)	
FEC ,n	3.0 (2.0 − 4.0)	2.0 (2.0 − 3.0)	0.490		3.0 (1.0 − 4.0)	2.75 (1.0 − 4.0)	0.446
DEC (n,%)			0.212				0.282
two-way	132 (78.6)	82 (73.9)			118 (88.7)	76 (82.6)	
forward	21(12.5)	22 (19.8)			9 (6.8)	12 (13.0)	
reverse	15 (8.9)	7 (6.3)			6 (4.5)	4(4.3)	
EMBLP (n,%)			0.574				0.607
Type I	27 (15.5)	19 (16.4)			32 (18.4)	23(19.8)	
Type II	78 (44.8)	58 (50.0)			75 (43.1)	55 (47.4)	
Type III	69 (39.7)	39 (33.6)			67(38.5)	38 (32.8)	
EMBLB (n,%)			0.009				0.001
<5	84 (48.3)(37.62%)	74 (63.8)			59(33.9)	71 (61.2)	
≥5	90 (51.7)	42 (36.2)			115 (66.1)	45 (38.8)	
EV (cm^3^)	2.87(2.17 − 3.80)	2.92 (2.30 − 3.74)		0.8862	3.09 (2.32 − 4.19)	3.11 (2.36 − 3.96)	0.880

Forward: Cervico-to-fundal movement. Reverse: fundal-to-cervical movement. Abbreviations: EMT endometrial thickness; EMP endometrial pattern; FEC frequency of endometrial contraction; DEC direction of endometrial contraction; EMBLP endometrial blood flow pattern; EMBLB endometrial blood flow branches; EV endometrial volume

### Changes in endometrial blood flow parameters

It was found that different pregnancy outcome samples did not show significant differences in the changes in endometrial blood flow pattern and blood flow branch between the day of progesterone initiation and the day before FET (Table 3).

**Table 3:** Comparison of changes in endometrial blood flow indices from the day of progesterone administration to the day before embryo transfer between pregnant and non-pregnant patients

Parameters	Pregnant group	Non-pregnant group	*P*-value
Number of patients	174	116	---
Change in EMBLP (n, %)			0.269
N-Change	148 (85.06)	93(80.17)	
Type I-II	3 (1.72)	5(4.31)	
Type I-III	1(0.57)	3(2.58)	
Type II-III	22 (12.64)	15(12.93)	
Change in EMBLB(n, %)			0.774
N-Change	134(77.01)	91 (78.45)	
Increment	40 (22.99)	25 (21.55)	

N-Change: no significant change in parameters at two time points. Type I-II: endometrial blood flow pattern from I on the day of progesterone administration to II on the day before embryo transfer. Increment: blood flow branches from < 5 on the day of transformation to ≥ 5 on the day before transfer. Abbreviations: EMBLP, endometrial blood flow pattern; EMBLB, endometrial blood flow branches

### Logistic regression and receiver operating characteristics curve analysis

Binary stepwise logistic regression analysis was performed to identify the covariates independently associated with the primary outcome of clinical pregnancy (Table 4). After adjusting for age, AFC, number of embryos transferred, embryo level, the proportion of good-quality embryo transfer, endometrial type, and endometrial blood flow branches, and changes of blood flow at two time points, only the endometrial blood flow branches on the day before embryo transfer significantly improved the chance of pregnancy, with ORs of 3.001 (95% CI: 1.448 − 6.219, P = 0.003).

**Table 4:** Logistic regression analysis to identify covariates independently associated with the clinical pregnancy

Covariates		Logistic regression coefficient (B)	Adjusted OR (95% CI)	*P*-value		
Age	-0.054		0.947 (0.891 − 1.007)	0.082		
AFC	0.032		1.033 (0.989 − 1.078)	0.147		
No. of embryos transferred	0.550		1.734 (0.975 − 3.083)	0.061		
Blastocysts transfer						
NO				Ref.		
YES	0.587		1.798 (0.990 − 3.266)	0.054		
Good quality embryo transfer						
NO				Ref.		
YES	0.010		1.010 (0.554 − 1.842)	0.974		
EMBLB on the endometrial transformation day						
< 5				Ref.		
≥ 5	0.184		1.202 (0.595 − 2.428)	0.608		
EMBLB on the day before embryo transfer						
< 5				Ref.		
≥ 5	1.099		3.001 (1.448 − 6.219)	0.003		
Change in EMBLB						
N-Change				Ref.		
Increment	0.497		1.643 (0.650 − 4.151)	0.294		

Abbreviations: AFC antral follicle; EMBLB endometrial blood flow branches; OR odds ratio; CI confidence interval

## Data Availability

Not applicable.

## Abbreviations

HRT-FET: Hormone Replacement Therapy-Frozen Embryo Transfer
IVF: In Vitro Fertilization
PD-US: Power Doppler Ultrasound
EMT: Endometrial Thickness
AFC: Antral Follicles
OR: Odds Ratio
CI: Confidence Interval
3D: Three-Dimensional
ETBF: Endometrial Tissue Blood Flow
EMP: Endometrial Pattern
FEC: Frequency of Endometrial Contraction
DEC: Direction of Endometrial Contraction
EMBLP: Endometrial Blood Flow Pattern
EMBLB: Endometrial Blood Flow Branches
EV: Endometrial Volume
